# Resting state fMRI data from subjects scanned with the EPI-PACE (Echoplanar Imaging – Prospective Acquisition CorrEction) sequence

**DOI:** 10.1016/j.dib.2018.01.089

**Published:** 2018-02-06

**Authors:** Pradyumna Lanka, Gopikrishna Deshpande

**Affiliations:** a AU MRI Research Center, Department of Electrical and Computer Engineering, Auburn University, Auburn, AL, USA; b Department of Psychological Sciences, University of California, Merced, CA, USA; c Department of Psychology, Auburn University, Auburn, AL, USA; d Alabama Advanced Imaging Consortium, Auburn University and University of Alabama Birmingham, AL, USA; e Center for Health Ecology and Equity Research, Auburn University, Auburn, AL, USA; f Center for Neuroscience, Auburn University, Auburn, AL, USA

## Abstract

Due to the confounding effects of head motion artifacts on resting-state functional connectivity (RSFC), there has been a growing interest in both acquisition and preprocessing strategies for removing motion-related artifacts from resting state functional Magnetic Resonance Imaging (Rs-fMRI) data. Prospective motion correction by the Siemens’ EPI-PACE sequence could offer new insights on the effectiveness of this sequence to correct head motion artifacts in Rs-fMRI data. The head motion parameters along with Rs-fMRI data obtained from 47 healthy individuals scanned with the EPI-PACE sequence is presented in this article. This data is useful to understand the effectiveness of prospective motion correction strategies such as 3D PACE for reducing head motion artifacts in Rs-fMRI data and help devise effective motion correction strategies. The utility of the EPI-PACE sequence in reducing motion correction artifacts in healthy controls can be found in our research article on the topic [1].

**Specifications Table**

**Table:** 

Subject area	*Brain imaging*
More specific subject area	*Head motion artifacts in resting state functional magnetic resonance imaging*
Type of data	*MRI Images, T-maps*
How data was acquired	*3T Verio MAGNETOM MRI Scanner (Siemens, Erlangen, Germany)*
Data format	*NifTi, MAT*
Experimental factors	*Resting- state fMRI data from 47 subjects were collected using the EPI PACE sequence.*
Experimental features	*The participants were scanned with the instructions to keep their eyes open, stay still and move their head as little as possible for the duration of the scans.*
Data source location	*Auburn, Alabama, USA*
Data accessibility	*Data for this article is can be found online at NITRC:*https://www.nitrc.org/projects/epi_pace_rest/

**Value of the data**

• Recent reports have shown that motion artifacts in resting state fMRI data have been under-appreciated [2], [3], [4]. Consequently, just performing rigid-body realignment of images and regression of realignment parameters is no longer considered enough to remove motion artifacts that may corrupt functional connectivity estimates. Even though the EPI-PACE (Prospective Acquisition CorrEction) sequence has been around for a decade [5], it needs to be re-evaluated in the context of resting-state fMRI functional connectivity and associated failure of retrospective motion-correction strategies to completely get rid of motion artifacts in the data.
• EPI-PACE is available as a Siemens product sequence and does not require any additional setup, such as external cameras which are typically used during prospective motion correction. Therefore, it is suitable for high throughput scanning in clinical settings as well.
• To the best of our knowledge, Rs-fMRI data using the PACE sequence is currently not available publicly. By releasing this data, we are hoping that it will be pooled with data from other sites for more robust analyses using larger sample sizes to gain a better understanding of the effects of head motion artifacts on functional connectivity by using prospective strategies such as EPI-PACE in combination with known retrospective strategies.
• The presence or absence of head motion artifacts can be compared with the data collected from EPI-PACE sequence to the normal EPI sequence for deciding on suitable acquisition protocols in hyperkinetic populations.
• The preprocessed data can be further used to test the efficacy of retrospective motion correction methods on data collected from the EPI-PACE sequence.

## 1 Data

This data release contains the following: (a) Defaced (i.e. facial features removed so that subjects’ faces cannot be reconstructed) T1 weighted MPRAGE anatomical images (b) 4D NifTi pre-processed images corresponding to resting-state functional magnetic resonance imaging (Rs-fMRI) data acquired using the EPI-PACE sequence, (c) files containing voxel-wise frame-wise displacement of the head (FDvox) for each subject, and (d) MATLAB files containing the motion metrics (3 translations and 3 rotations) and summary motion statistics for 47 healthy individuals. We provide the 4D NifTi data for several combinations of nuisance signal regressors and standard retrospective motion correction approaches: (i) CSF (cerebro-spinal fluid)+WM (white matter) signal regression, (ii) CSF+WM+GS (global signal) regression, (iii) CSF+WM+Friston-24 motion regression [6], (iv) CSF+WM+GS+Friston-24 motion regression, (v) CSF+WM+Friston-24 motion regression+motion censoring (FD_Power_ threshold >0.5 mm and 1 back and 2 forward volumes censored from the model) [3], and (vi) CSF+WM+GS+Friston-24 motion regression+motion censoring. We also provide a T-maps showing significant relationships between head motion (FD_vox_) and the BOLD timeseries for each of the 6 combinations as 3D NifTi images (.nii).

## 2 Experimental design, materials and methods

### 2.1 Participants

47 healthy adult participants (20 males/27 females, age 25.1±5 years) were recruited for this study. The scanning instructions for the subjects were to relax, keep their eyes open and head as still as possible during the scans. We provided head support and padding to ensure that the subjects were comfortable and the head motion was minimized. Informed consent of the subjects was obtained, and the scanning procedure was approved by the Institutional Review Board (IRB) at Auburn University.

### 2.2 Acquisition procedure

The subjects were scanned with a Siemens 3T MAGNETOM Verio scanner (Siemens Healthcare, Erlangen, Germany) using an EPI–PACE sequence with a 32 channel head coil. The acquisition parameters for the Rs-fMRI data are TR of 1000 ms, TE of 29 ms, Flip Angle of 90° with 16 slices, matrix=64×64, voxel size=3.5×3.5×5 mm^3^, Number of time points=250 for 12 subjects, 500 for 22 subjects and 1000 for 13 subjects. T1 weighted MPRAGE anatomical images with parameters of TE=2 ms, TR=1900 ms and 176 slices with 1×1×1 mm^3^ voxel size were acquired along with the functional images for aiding in the preprocessing of the functional images. The anatomical images were defaced by removing the facial features to protect the privacy of the subjects [7].

### 2.3 Preprocessing of the Rs-fMRI data

Standard Rs-fMRI preprocessing of the data was done using a MATLAB (MathWorks, Natick, USA) toolbox called Data Processing Assistant for Resting-State fMRI (DARPSF) toolbox [8]. The preprocessing pipeline included: removal of first five time points, slice timing correction and realignment and removal of linear and quadratic trends from the time series. Mean WM and CSF, GS and the Friston-24 parameters were regressed from the resting state fMRI time series. Motion corrupted volumes (FD_Power_ threshold >0.5 mm) along with 1 back and 2 forward volumes were censored from the data.

### 2.4 Calculation of head motion parameters

The DPRARSF toolbox outputs a MAT file and several text files which contains the head motion information from the subject, including relative displacement as defined by Framewise displacement (FD) and the absolute displacement as Total displacement (TD). The MAT-file includes FD_FSL_ [9], FD_Power_ [2], and FD_VanDijk_ [10]. The above measures are volumentric motion measures which assign a single motion value for all voxels across the brain for every time point. Since in PACE, slice positioning is adjusted before the acquisition of every volume, these realignment parameters, and the FD metrics are a measure of the residual motion (which was not corrected by PACE) rather than the actual motion of the head. DPARSF also calculates voxel-specific head motion metrics such as voxel-specific framewise displacement (FD_vox_) which considers the displacement of every voxel over time [3], [4], and this information has also been provided.

### 2.5 T-maps showing significant motion-BOLD relationships

The Rs-fMRI BOLD signal was pre-processed with several combinations of nuisance signal regressors and motion correction strategies: (i) CSF+WM regression, (ii) CSF+WM+GS regression, (iii) CSF+WM+Friston-24 motion regression, (iv) CSF+WM+GS+Friston-24 motion regression, (v) CSF+WM+Friston-24 motion regression+motion censoring (FD_Power_ threshold >0.5 mm and 1 back and 2 forward volumes censored from the model), and (vi) CSF+WM+GS+Friston-24 motion regression+motion censoring. For each of the above combinations of retrospective processing strategies, the Pearson's correlation coefficient between the time series of voxel-specific framewise displacement (FD_vox_) and the BOLD signal was calculated for every voxel in the brain. This was followed by the Fisher's r to z transformation. The resultant z -maps were then normalized to the standard MNI template (3 mm^3^ cubic voxels). A one-sample t-test was performed on the normalized correlation maps with a significance level of *p*<0.05 (FDR corrected) to give the T-maps presented with this paper. These T-maps can be used to detect consistent patterns of motion-BOLD relationships. More information about the data be obtained from Lanka et al. [1].
